# Stem canker pathogen *Botryosphaeria dothidea* inhibits poplar leaf photosynthesis in the early stage of inoculation

**DOI:** 10.3389/fpls.2022.1008834

**Published:** 2022-09-20

**Authors:** Junchao Xing, Min Li, Jinxin Li, Wanna Shen, Ping Li, Jiaping Zhao, Yinan Zhang

**Affiliations:** Institute of Ecological Conservation and Restoration, Chinese Academy of Forestry, Beijing, China

**Keywords:** poplar stem canker, fungal pathogens, *Botryosphaeria dothidea*, photosynthesis, stomatal closure, chlorophyll fluorescence

## Abstract

Fungal pathogens can induce canker lesions, wilting, and even dieback in many species. Trees can suffer serious physiological effects from stem cankers. In this study, we investigated the effects of *Botryosphaeria dothidea* (*B*. *dothidea*) on *Populus bolleana* (*P. bolleana*) leaves photosynthesis and stomatal responses, when stems were inoculated with the pathogen. To provide experimental and theoretical basis for preventing poplar canker early. One-year-old poplar stems were inoculated with *B*. *dothidea* using an epidermal scraping method. In the early stage of *B*. *dothidea* inoculation (2–14 days post inoculation, dpi), the gas exchange, stomatal dynamics, hormone content, photosynthetic pigments content, chlorophyll fluorescence parameters, and non-structural carbohydrate (NSC) were evaluated to elucidate the pathophysiological mechanism of *B*. *dothidea* inhibiting photosynthesis. Compared with the control groups, *B*. *dothidea* noteworthily inhibited the net photosynthetic rate (*P*_n_), stomatal conductance (*G*_s_), intercellular CO_2_ concentration (*C*_i_), transpiration rate (*T*_r_), and other photosynthetic parameters of poplar leaves, but stomatal limit value (*L*_s_) increased. Consistent with the above results, *B*. *dothidea* also reduced stomatal aperture and stomatal opening rate. In addition, *B*. *dothidea* not only remarkably reduced the content of photosynthetic pigments, but also decreased the maximum photochemical efficiency (*F*_v_/*F*_m_), actual photochemical efficiency (*Φ*_PSII_), electron transfer efficiency (ETR), and photochemical quenching coefficient (*q*_P_). Furthermore, both chlorophyll and *Φ*_PSII_ were positively correlated with *P*_n_. In summary, the main reason for the abated *P*_n_ under stem canker pathogen was that *B*. *dothidea* not merely inhibited the stomatal opening, but hindered the conversion of light energy, electron transfer and light energy utilization of poplar leaves. In general, the lessened CO_2_ and *P*_n_ would reduce the synthesis of photosynthetic products. Whereas, sucrose and starch accumulated in poplar leaves, which may be due to the local damage caused by *B*. *dothidea* inoculation in phloem, hindering downward transport of these products.

## Introduction

Plant photosynthesis is continuously challenged by a variety of environmental stresses, including abiotic stresses such as drought, high temperature, salt, and UV radiation, as well as biotic stresses caused by fungal pathogens and pests (Major et al., 2010; Suzuki et al., 2014; Bernal et al., 2015; Gururani et al., 2015; Ruehr et al., 2016; Saiki et al., 2017; Fermín et al., 2018; Blackman et al., 2019; Savi et al., 2019). Tree canker is a kind of most serious tree bark disease occur mainly in the stems and branches which have various kinds of pathogens, wide distribution of host and complex symptoms (Slippers and Wingfield, 2007; Marsberg et al., 2017). The canker disease of royal poinciana (*Delonix regia*) can result from fungal pathogen *Neoscytalidium dimidiatum* attacking different parts of the plant under certain favorable conditions (Raish et al., 2020). Fungal pathogens cause bark blisters and sunken lesions on stems in by first destroying the phloem and cambium (Biggs et al., 1983; Mcpartland and Schoeneweiss, 1984; Czemmel et al., 2015). Canker caused by necrotrophic fungal pathogens is commonly forming necrotic lesions be limited to the regions around the infection sites and do not girdle the branches pathologically at once, however, canker lesions on saplings or cankers abundant and/or perennial persistently can girdle branches, which eventually lead to yellowing tree canopy withering, dieback distal to the canker region, and even death (Marsberg et al., 2017; Li et al., 2019; Xing et al., 2020). However, the mechanisms of stem canker inhibit photosynthesis in distal leaves need more in-depth investigation.

Fungal pathogen infection usually results in plant tissue necrosis, a significant reduction in net photosynthetic rate (*P*_n_) and carbon assimilation, slower growth, and even death (Berger et al., 2007; Moradi and Ismail, 2007; Christian et al., 2008). Leaf pathogens can directly depress photosynthesis by regulating stomatal opening, decreasing gas exchange, impairing green tissues, damaging photosynthetic apparatus, and suppressing key enzyme activities (Gruber et al., 2012; Júnior et al., 2017; Fermín et al., 2018; Franziska et al., 2018). Globally, *B*. *dothidea* causes canker, dieback, shoot blight, and fruit rot (Marsberg et al., 2017). Studies have also shown that stem canker can remotely alter the photosynthetic characteristics of distal leaves (Rohrs-Richey et al., 2011; Cerqueira et al., 2017). *Quambalaria coyrecup* remarkably reduced *Corymbia calophylla* leaf net *P*_n_ and stomatal conductance in the later stage of inoculation (Hossain et al., 2019). In the interaction between hazelnut (*Corylus avellana* L.) and the canker pathogen *Anisogramma anomala*, canopy branch blight occurs distal to the canker areas (Lachenbruch and Zhao, 2019). In the early stage of poplar inoculation with stem canker pathogen *B*. *dothidea* and *Valsa sordida* (*V*. *sordida*), net *P*_n_ and stomatal conductance were prominently weakened (Li et al., 2019; Xing et al., 2020). The above remarks indicate that although plant canker occurs on stems and branches, it eventually affects the photosynthetic process of distal leaves. In their early stages, canker diseases are unclear in terms of their physiology.

Stomata can respond rapidly to environmental stress, regulating gas exchange, controlling CO_2_ uptake and water loss by adjusting stomatal size, thereby affecting photosynthesis (Schroeder et al., 2001; Melotto et al., 2006; Lawson and Vialet-Chabrand, 2019). A part of the immune responses of plants is closing stomata against pathogenic microbes (McLachlan et al., 2014). Alternatively, persistent stomatal closure leads to the reduced CO_2_ uptake, affecting plant photosynthetic productivity, eventually depleting carbohydrates in plant tissues (O′Grady et al., 2013). Pathogens also interfere with phytohormones metabolism (Bari and Jones, 2009). In one sense, hormone promote disease development, but they also participate in plant immune responses, which increases plant resistance (Bari and Jones, 2009; Verma et al., 2016; Kunkel and Harper, 2018). Abscisic acid (ABA), jasmonic acid (JA), and auxin (IAA) are involved in stomatal movement. Research has shown that stress promotes crosstalk between phytohormones (Ku et al., 2018). Necrotrophic pathogens produce a variety of hormone-like active substances during the infection period, which directly or indirectly affect hormone metabolism in plants (Yang et al., 2015).

Biotic stress can disrupt plant photosynthetic apparatus (Júnior et al., 2017). Chlorophyll fluorescence parameters can sensitively reflect the activity of the photosynthetic apparatus (Björkman and Demmig, 1987; Krause and Weis, 1991; Watling et al., 2000). The leaf maximum photochemical efficiency (*F*_v_/*F*_m_) was strikingly reduced in poplar trees infected by the leaf rust fungus, *Melampsora medusae* (Fermín et al., 2018). As well as leaf pathogens, stem pathogen, *Seiridium cardinale*, also inhibited leaf photosystem II (Muthuchelian et al., 2005). *Botrytis cinerea* obviously reduced strawberry leaf *F*_v_/*F*_m_ and chlorophyll index (Meng et al., 2020). In pecans infected with stem canker agents (*Phomopsis* spp.), the leaf chlorophyll fluorescence parameters declined with the extension of infection time (Guillermo et al., 2021).

In this study, we recruited the *P*. *bolleana*-*B*. *dothidea* interaction system as a research object. In the early stage of *B*. *dothidea* inoculation, the gas exchange, stomatal dynamics, hormone content, photosynthetic pigments content, and chlorophyll fluorescence parameters were measured. Through analyzing the effects of *B*. *dothidea* on leaf stomatal movement, light energy utilization, and electron transfer, the pathophysiological mechanism of poplar stem canker inhibiting the leaf photosynthesis was investigated, aiming to provide a theoretical basis on the pathogenic mechanism operating at the initial stage of poplar canker.

## Materials and methods

### Plant material, fungal pathogen, and inoculation

One-year-old *P*. *bolleana* clones from cuttings were planted in 5-L pots containing sand. Potted plants were placed within the greenhouse at Chinese Academy of Forestry and watered 2–3 times weekly at 20–25°C under 12-h daily photoperiod with 200–300 μmol m^–2^s^–1^. The *P*. *bolleana* saplings were assigned to the two groups: inoculation with *B*. *dothidea* (Bd) and potato dextrose agar (CTR). *P*. *bolleana* leaves (4th–6th mature leaves from the top) were selected for the following measurements. Seven biological replicates in every group.

Stems infected with *B. dothidea* were sampled from *Populus hopeiensis* Hu et Chow in Yi County, Hebei Province, China. Infected regions were cut into small pieces and disinfested with 75% ethanol for 30–60 s, then transferred onto potato dextrose agar (PDA, 2.0% potato extract, 2.0% dextrose, and 1.5% agar; pH 6.0). Following hyphal tip purification, all isolates were plated on fresh PDA dishes for further analysis. A variety of isolates were tested for pathogenicity by inoculating stems on poplar saplings with mycelium. The most pathogenic isolate was identified (NCBI accession number: MK990559 for rRNA-ITs and MN025271 for EF1α gene) and stored at 4°C on PDA plates in Chinese academy of forestry.

Inoculation treatments were performed as following steps. Briefly, after cultivation on PDA for 7 days, the mycelium of *B*. *dothidea* was cut into strips with 1.2–1.5 cm in width and 2.5–3.0 cm in length, which were used to inoculate *P*. *bolleana* stems. PDA culture medium used as control. The inoculation sites (30 cm above the sand surface) on *P*. *bolleana* stems were pre-sterilized with 75% ethanol, then the barks were scraped gently with blade. Taking care to avoid damaging the remaining phloem, cambial and xylem tissue during the scraping process. The scraped region was covered completely by strips of *B*. *dothidea* mycelium or PDA medium. Finally, all strips were wrapped with sterilized Parafilm™.

### Photosynthetic parameters

Net *P*_n_, stomatal conductance (*G*_s_), intercellular CO_2_ concentration (*C*_i_), transpiration rate (*T*_r_), and vapor pressure deficit (VPD) were measured using a Li-6400XT portable photosynthesis system (LI-COR, Lincoln, USA). The selected *P. bolleana* leaves were measured and recorded from 9:00 to 11:00 a.m. on 0, 2, 4, 6, 8, 10, 14 dpi. Stomatal limit value (*L*_s_) and water use efficiency (WUE) were calculated using the following formulae:


$$L_{\text{s}}=1-C_{\text{i}}/C_{\text{a}}\,\left({\text{CO}}_{2}\,\mathrm{concentration}\,\mathrm{in}\,\mathrm{the}\,\mathrm{air}\right)$$

$$\text{WUE}=P_{\text{n}}/T_{\text{r}}$$

### Stomatal movement

#### Stomatal density

The lower epidermis of *P*. *bolleana* leaves was examined by SEM (5136, TESCAN, Brno, CS) to capture images. 10–15 visual fields were selected randomly at the magnification of 1 kx and 2 kx, respectively. Stomatal density of 6 and 14 dpi was calculated as the number of stomata per unit leaf area (mm^2^).

#### Stomatal aperture and opening rate

*P*. *bolleana* leaves of 6 and 14 dpi were washed 3–4 times with PBS buffer. Preparation for the samples was carried out according to Zhu et al. (2019). The resulting samples were examined through scanning electron microscopy (SEM, 5136, TESCAN, Brno, CS). Image-Pro-Plus 6.0 software was used to measure the stomatal aperture and opening rate. The stomatal aperture was evaluated by measuring the width of the stomatal pore observed under the SEM.

### Plant hormone content determination

*P. bolleana* leaves of 6 and 14 dpi were collected. All leaves were ground into powder in liquid nitrogen, then 0.3 g was taken and stored at 4°C for the following experiment. Plant endogenous hormone content was analyzed by Enzyme-Linked Immunosorbent Assay (ELISA) according to Yang et al. (2001).

### Photosynthetic pigments content

The acetone extraction and colorimetric assays were applied to determine the content of chlorophyll a (Chl a), chlorophyll b (Chl b), and carotenoids (Car). *P*. *bolleana* leaves of 6 and 14 dpi were minced by scissors. A 0.2 g sample was accurately weighed and 20 mL of 80% acetone was added to extract photosynthetic pigments in the dark for 48 h. Absorbance was, respectively, read at 663, 645, and 470 nm using a spectrophotometer (Dynamax, CA, US). The following formulae were used for calculation of photosynthetic pigments content:


$$\mathrm{Chl}\,a=12.21\,A_{663}-2.81\,A_{645}$$

$$\mathrm{Chl}\,b=20.13\,A_{645}-5.03\,A_{663}$$

$$\text{Car}=\left(1000\,A_{470}-3.27\,\mathrm{Chl}\,a-104\,\mathrm{Chl}\,b\right)/229$$

Pigmentscontent(mg/g)=[pigmentsconcentration(mg/L)×extractsvolume(ml)×dilutionmultiple]/samplequality(g)

### Chlorophyll fluorescence parameters

Chlorophyll fluorescence parameters were measured with FMS-2 Pulse Modulated Fluorometer (FMS-2, Hansatech, UK). *P*. *bolleana* leaves of 0, 2, 4, 6, 8, 10, and 14 dpi were dark-adapted for 30 min, then the initial fluorescence (*F*_0_), maximum fluorescence (*F*_m_), and maximum photochemical efficiency of PSII (*F*_v_/*F*_m_) were determined in darkness. After light-adaption for 30 min, the initial fluorescence (*F’*_o_), maximum fluorescence (*F’*_m_), steady-state fluorescence (*F*_s_), and actual PSII efficiency (*Φ*_PSII_) were measured under irradiance. After that, *F*_v_/*F*_m_, *Φ*_PSII_, electron transfer rate (ETR), photochemical quenching coefficient (*q*_P_), and non-photochemical quenching coefficient (NPQ) were calculated according to the following formulae:


$$F_{\text{v}}/F_{\text{m}}=\left(F_{\text{m}}-F_{0}\right)/F_{\text{m}}$$

$$\Phi_{\text{PSII}}=\left(F_{\text{m}}^{\prime }-F_{\text{s}}\right)/F_{\text{m}}^{\prime }$$

$$\text{ETR}=\Phi_{\text{PSII}}\times 0.5\times 0.84$$

$$q_{\text{P}}=\left(F_{\text{m}}^{\prime }-F_{\text{s}}\right)/\left(F_{\text{m}}^{\prime }-F_{0}^{\prime }\right)$$

$$\text{NPQ}=\left(F_{\text{m}}-F_{\text{m}}^{\prime }\right)/F_{\text{m}}^{\prime }$$

### Non-structural carbohydrate concentration assay

*P*. *bolleana* leaves of 6 and 14 dpi were sampled and promptly frozen in liquid nitrogen and stored at –80°C until assay. The extraction and *C*_i_ of starch and sucrose were measured using the Plant Sucrose Sugars Assay Kit and Soluble Starch Assay Kit (BC2465 and BC0705; Solarbio Life Sciences, Beijing, China) according to the manufacturer’s instructions.

### Statistical analysis

All of the experimental data were subjected to SPSS version 17.0 for statistical tests and analyses. *P* < 0.05 was considered significant unless otherwise notes. All statistical data were presented as mean ± standard error. In this study, correlation analysis was performed on Chl, *Φ*_PSII_ and *P*_n_, and linear regression fitting was performed. All fittings were tested by *t*-test (*P* < 0.001).

## Results

### Photosynthetic parameters

**FIGURE 1:**
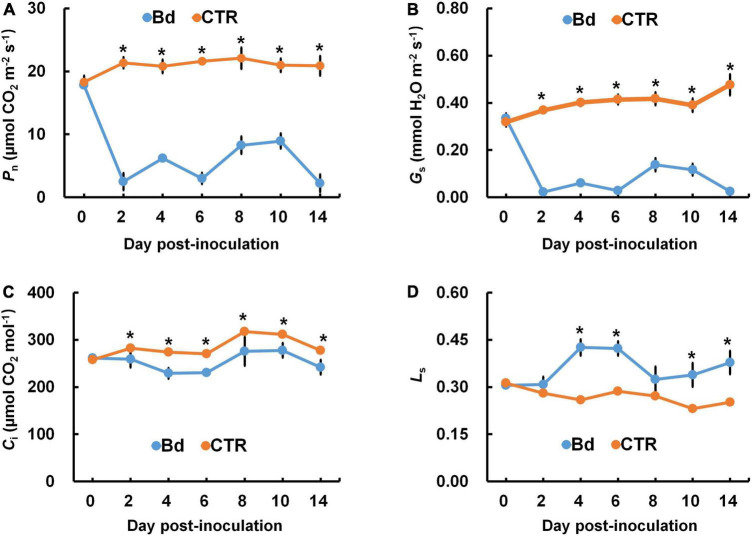
*B*. *dothidea* inoculation on photosynthetic parameters in *P*. *bolleana* leaves. One-year old poplar stems were inoculated, respectively, by *B*. *dothidea* (Bd) or PDA (CTR). **(A)** Net photosynthetic rate (*P*_n_), **(B)** stomatal conductance (*G*_s_), **(C)** intercellular CO_2_ concentration (*C*_i_), and **(D)** limiting value of stomata (*L*_s_) were measured at 0, 2, 4, 6, 8, 10, and 14 dpi. Data are presented as the mean of seven replicates. Error bars represent the standard error of the mean. Asterisks denote significant difference at *P* < 0.05 between treatments.

**FIGURE 2:**
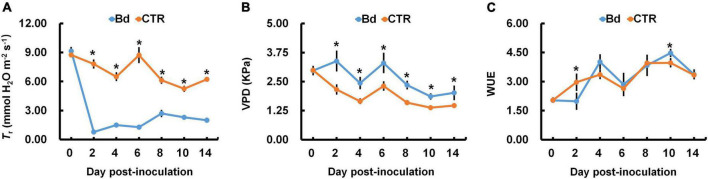
*B*. *dothidea* inoculation on water transportation in *P*. *bolleana* leaves. One-year old poplar stems were inoculated, respectively, by *B*. *dothidea* (Bd) or PDA (CTR). **(A)** Transpiration rate (*T*_r_), **(B)** vapor pressure deficit (VPD), and **(C)** water use efficiency (WUE) were measured at 0, 2, 4, 6, 8, 10, and 14 dpi. Data are presented as the mean of seven replicates. Error bars represent the standard error of the mean. Asterisks denote significant difference at *P* < 0.05 between treatments.

The photosynthetic parameters were measured in leaves of *B*. *dothidea*-inoculated and control poplars. *P*_n_ and *G*_s_ declined rapidly in *B*. *dothidea* inoculation treatments (range: 2–14 dpi, Figures 1A,B). On the fourth day after *B*. *dothidea* inoculation, *P*. *bolleana* leaves displayed completely opposite trends in *C*_i_ and *L*_s_ (Figures 1C,D). *C*_i_ level declined after *B*. *dothidea* inoculation (range: 4–14 dpi), while *L*_s_ showed an increasing trend. These results suggested that the main reason for declined *P*_n_ after *B*. *dothidea* inoculation was stomatal restriction. In addition, compared with the control plants, *T*_r_ of inoculated plants was markedly reduced, VPD increased, but WUE had no significant change, indicating that *B*. *dothidea* did not cause damage to the water transport in the early stage of inoculation (Figures 2A–C).

### Stomatal behavior

**FIGURE 3:**
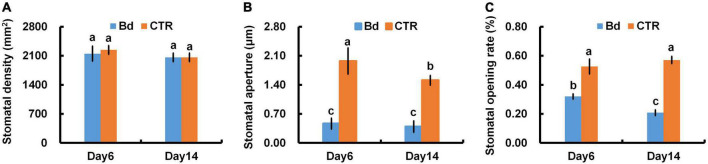
*B*. *dothidea* inoculation on stomatal movement in *P*. *bolleana* leaves. One-year old poplar stems were inoculated, respectively, by *B*. *dothidea* (Bd) or PDA (CTR). Sample collection and stomatal movement parameters measurement were performed at 6 and 14 dpi. **(A)** Stomatal density, **(B)** stomatal aperture, **(C)** stomatal opening rate. Each column is the mean of seven replicates. Error bars represent the standard error of the mean. Columns labeled with different letters (a–c) denote a significant difference (*P* < 0.05) between treatments.

**FIGURE 4:**
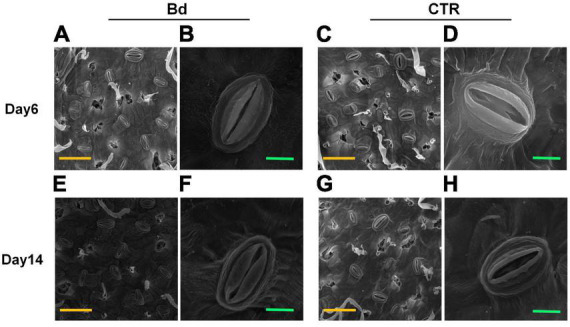
*B*. *dothidea* inoculation on stomatal characteristics in *P*. *bolleana* leaves. Representative images of stomatal characteristics under poplar stems inoculated by *B*. *dothidea* (Bd) or PDA (CTR) at 6 and 14 dpi **(A**–**H)**. **(B,D,F,H)** Showed the enlarged images of stomatal characteristics. Yellow scale bars **(A,C,E,G)** = 50 μm. Green scale bars **(B,D,F,H)** = 5 μm.

At 6 and 14 dpi, leaves of *B. dothidea*-inoculated and control poplars showed any difference in stomatal density (Figure 3A), which may be associated with the detectable leaves having already maturated. *B*. *dothidea* inoculation on poplar stem could inhibit stomatal opening of distal leaves at 6 and 14 dpi (Figures 4A,B,E,F). In controls, the stomata were almost completely opened (Figures 4C,D,G,H). Nevertheless, compared with control plants, *B*. *dothidea* inoculation reduced the width of the stomatal aperture by 76.1% at 6 dpi and 74.2% at 14 dpi (Figure 3B). Accordingly, the stomatal opening rate of *B*. *dothidea*-inoculated plants was also decreased by 39.6% at 6 dpi than in comparison with controls, 63.2% at 14 dpi (Figure 3C). The above results were combined with the declined *G*_s_ and increasing *L*_s_ (Figures 1B,D).

### Variation in hormone

**FIGURE 5:**
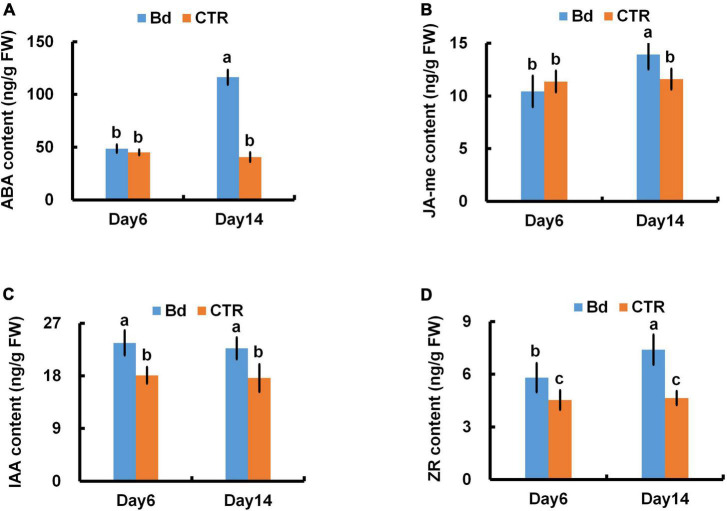
*B*. *dothidea* inoculation on phytohormone content in *P*. *bolleana* leaves. One-year old poplar stems were inoculated, respectively, by *B*. *dothidea* (Bd) or PDA (CTR). Sample collection and phytohormone content measurement were performed at 6 and 14 dpi. **(A)** ABA content, **(B)** JA-me content, **(C)** IAA content, **(D)** ZR content. Each column is the mean of seven replicates. Error bars represent the standard error of the mean. Columns labeled with different letters (a–c) denote a significant difference (*P* < 0.05) between treatments.

Stomatal closure is usually controlled by hormone. At 6 dpi, the content of ABA and JA-me in leaves of *B*. *dothidea*-inoculated plants had no significant difference with the controls (Figures 5A,B). Whereas, at 14 dpi, the level of ABA and JA-me in leaves of *B*. *dothidea*-inoculated plants, respectively, increased by nearly twofold and 24.6% in comparison with their controls (Figures 5A,B). In contrast with controls, *B*. *dothidea*-inoculated plants sustained higher content of IAA and zeatin (ZR) across the whole observation (Figures 5C,D). Compared with the control plants, IAA content increased by 30.6 and 28.9%, while ZR increased by 28.3 and 59.5% at 6 and 14 dpi, respectively (Figures 5C,D).

### Photosynthetic pigments content

**FIGURE 6:**
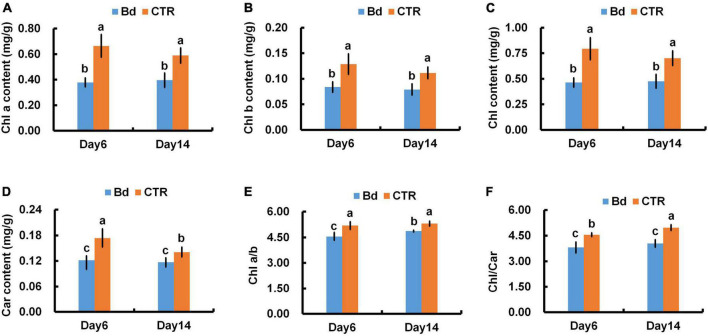
*B*. *dothidea* inoculation on photosynthetic pigments in *P*. *bolleana* leaves. One-year old poplar stems were inoculated, respectively, by *B*. *dothidea* (Bd) or PDA (CTR). Sample collection and photosynthetic pigments measurement were performed at 6 and 14 dpi. **(A)** Chlorophyll a content (Chl a), **(B)** Chlorophyll b content (Chl b), **(C)** total Chlorophyll content (Chl), **(D)** Carotenoids content (Car). **(E)** Chlorophyll a/b ratio, (Chl a/b), **(F)** Chlorophyll/Carotenoids ratio, (Chl/Car). Each column is the mean of seven replicates. Error bars represent the standard error of the mean. Columns labeled with different letters (a–c) denote a significant difference (*P* < 0.05) between treatments.

As a result of *B*. *dothidea* inoculation, chlorophyll content, fractional chlorophyll a, b, and carotenoids content were severely reduced in poplar leaves at different degrees (Figures 6A–D). At 6 dpi, in contrast with control leaves, *B*. *dothidea* inoculation caused a 42.4, 38.5, 41.8, 29.4, 12.2, and 16.3% decline in Chl a, Chl b, total chlorophyll (Chl), Car, chlorophyll a/b (Chl a/b) and total chlorophyll/carotenoids (Chl/Car), respectively (Figure 6). Compared to control leaves, Chl a content decreased by 32.2% at 14 dpi under *B*. *dothidea* inoculation, Chl b by 27.3%, Chl by 31.4%, Car by 14.3%, Chl a/b by 8.1%, and Chl/Car by 18.5% (Figure 6).

### Evaluation of chlorophyll fluorescence characteristics

**FIGURE 7:**
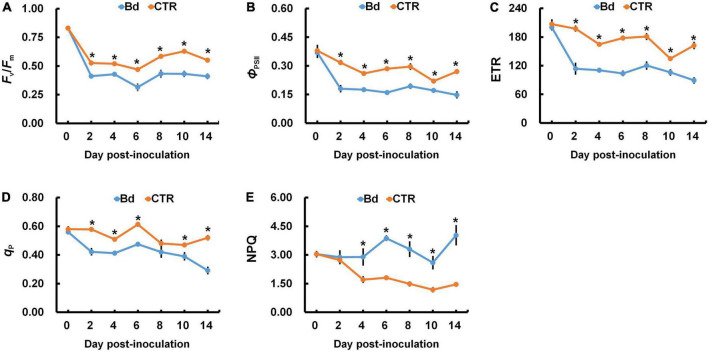
*B*. *dothidea* inoculation on chlorophyll fluorescence parameters in *P*. *bolleana* leaves. One-year old poplar stems were inoculated, respectively, by *B*. *dothidea* (Bd) or PDA (CTR). Chlorophyll fluorescence parameters were measured at 0, 2, 4, 6, 8, 10, and 14 dpi. **(A)** Ratio of variable to maximal chlorophyll fluorescence (*F*_v_*/F*_m_), **(B)** Actual photochemical efficiency of PSII (*Φ*_PSII_), **(C)** Electron transfer rate (ETR), **(D)** Photochemical quenching coefficient (*q*_P_). **(E)** Non-photochemical quenching coefficient (NPQ). Data are presented as the mean of seven replicates. Error bars represent the standard error of the mean. Asterisks denote significant difference at *P* < 0.05 between treatments.

When poplar stems were inoculated with *B*. *dothidea*, the chlorophyll fluorescence parameters of leaves were seriously affected. Plants inoculated with *B*. *dothidea* experienced abrupt declines in *F*_v_/*F*_m_, *Φ*_PSII_, and ETR, which were consistently lower than those in control plants (Figures 7A–C). Additionally, *B*. *dothidea*-inoculated poplars had less *q*_P_ than controls, except for 8 dpi (Figure 7D). The undifferentiated data at 8 dpi did not affect the whole variant trend of *q*_P_. Compared with control plants, a higher NPQ was always observed after *B*. *dothidea* inoculation from the fourth day onward (Figure 7E).

### Correlation analysis in physiological indices

**FIGURE 8:**
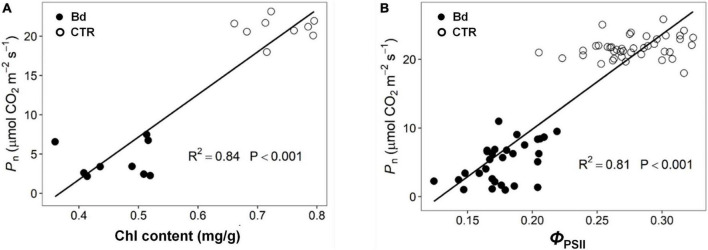
Correlation analysis of parameters. **(A)** Chlorophyll content (Chl) Vs. net photosynthetic rate (*P*_n_), **(B)** actual photochemical efficiency (*Φ*_PSII_) Vs. net photosynthetic rate (*P*_n_).

According to the linear correlation performed, both Chl and *Φ*_PSII_ were positively correlated with *P*_n_ throughout *B*. *dothidea* inoculation (Figure 8).

### Leaf non-structure carbohydrate content

**FIGURE 9:**
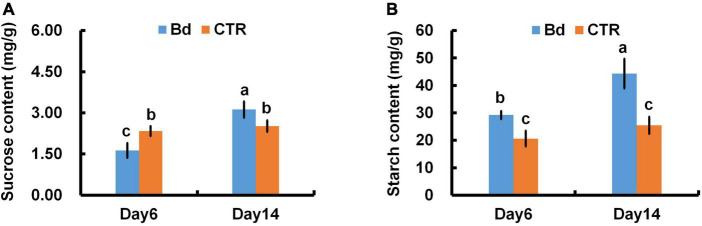
*B*. *dothidea* inoculation on sucrose and starch content in *P*. *bolleana* leaves. One-year old poplar stems were inoculated, respectively, by *B*. *dothidea* (Bd) or PDA (CTR). Sample collection, sucrose and starch content measurements were performed at 6 and 14 dpi. **(A)** Sucrose content, **(B)** starch content. Each column is the mean of seven replicates. Error bars represent the standard error of the mean. Columns labeled with different letters (a–c) denote a significant difference (*P* < 0.05) between treatments.

*B*. *dothidea*-inoculated plants showed similar trends in starch and sucrose content of distal leaves. At 6 dpi, *B*. *dothidea* inoculation plants contained less sucrose than controls (Figure 9A). With the prolonged inoculation time, more sucrose was accumulated in *B*. *dothidea*-inoculated plants at 14 dpi (Figure 9A). In *B*. *dothidea*-inoculated plants, starch content was 48.5 and 80.5% higher than in controls at 6 and 14 dpi, respectively (Figure 9B).

## Discussion

### Photosynthetic rate

Photosynthesis can negatively affect by leaf pathogens in several ways, including modulating stomatal opening, reducing gas exchange, damaging green tissues and photosynthetic apparatus (Gruber et al., 2012; Júnior et al., 2017; Fermín et al., 2018; Franziska et al., 2018). Additionally, stem canker fungi such as *Quambalaria coyrecup* and *Anisogramma anomala* are among those may alter the photosynthetic properties of distal leaves remotely on *Corymbia calophylla* and hazelnut (Hossain et al., 2019; Lachenbruch and Zhao, 2019). In previous studies, we had similar results that leaves in *B*. *dothidea*-inoculated and *V*. *sordida*-inoculated plants showed weakened *P*_n_ and *G*_s_ during the observation stages (Li et al., 2019; Xing et al., 2020). In the initial stage of stress, stomatal closure resulted in the reduction in carbon assimilation thus reducing net photosynthesis. This phenomenon is known as a stomatal limitation. The type of stomatal limitation is mainly determined by *C*_i_ and *L*_s_ (Farquhar and Sharkey, 1982). In this study, *B*. *dothidea* inoculation obviously declined *P*_n_ and *G*_s_ at early inoculation stages (Figures 1A,B). *C*_i_ level drastically reduced at 2–14 dpi, while *L*_s_ showed an opposite trend, indicating that *P*_n_ is reduced by stomatal limitation at the early stage of inoculation (Figures 1C,D). Stem canker pathogens have been shown to infiltrate xylem tissues in histopathological studies (Biggs et al., 1983; Mcpartland and Schoeneweiss, 1984; Czemmel et al., 2015). Fungal pathogens also reduce plant hydraulic conductance and sap flow (Parke et al., 2007; Hossain et al., 2019). In this study, the reduced *G*_s_ also dramatically reduced the *T*_r_ when compared with control treatments (Figure 2A). Conversely, VPD was increased, and WUE had no significant change, indicating that *B*. *dothidea* inoculation did not cause severe damage to water transport during the early stage (Figures 2B,C).

### Stomatal closure

Stomatal movement is affected by guard cells’ osmotic/turgor pressure, the latter reacting in response to external and internal stimuli (Blatt, 2000). The stomata can adjust their size in response to environmental stress, which regulates gas exchange, CO_2_ uptake and water loss (Schroeder et al., 2001; Melotto et al., 2006; Lawson and Vialet-Chabrand, 2019). Plants close their stomata to combat pathogenic microbes as part of their immune response (McLachlan et al., 2014). In this study, *B*. *dothidea* inoculation on poplar stems induced stomatal closure in distal leaves (Figure 4). Although *B*. *dothidea* inoculation caused remarkable reduction of stomatal aperture and opening rate, no obvious influence was detected on stomatal density (Figure 3), which may be related to the maturation of the detected leaves. These results were consistent with the decreased *G*_s_ and *C*_i_, as well as the increased *L*_s_ (Figures 1B,C). Photosynthesis is also reduced due to this phenomenon, which confirms stomatal limitation during *B*. *dothidea* inoculation (Li et al., 2019; Xing et al., 2020). We speculated that *B*. *dothidea* inoculation reduced the stomatal apertures and stomatal opening rate, which in turn limited the *G*_s_ subsequently caused a declined *P*_n_.

Phytohormones metabolism can also be disrupted by pathogens (Bari and Jones, 2009). Hormones are considered part of the immune system of plants, and may play a role in disease development (Verma et al., 2016; Kunkel and Harper, 2018). Similar to ABA, JA-me can also induce stomatal closure. There has been evidence that stress can lead to crossover of phytohormone in plant (Ku et al., 2018). ABA and JA-me exhibit phytohormones crosstalk in the pathway for stomatal closure (McLachlan et al., 2014). In this study, *B*. *dothidea* inoculation triggered stomatal closure at 6 and 14 dpi, but the level of ABA and JA-me only increased at 14 dpi (Figures 5A,B). In addition, IAA and ZR content in *B*. *dothidea*-inoculated plants were significantly higher than controls at 6 and 14 dpi (Figures 5C,D). It is generally believed that IAA and ZR contribute to the stomatal opening and inhibited ABA-induced stomatal closure (Blackman and Davies, 1984; Irving et al., 1992), which is in disagreement with our study. Pathogens that cause necrotroptosis produce diverse hormone-like substances and secondary metabolites, which disturb hormone metabolism and trigger other effects on distal tissues from the inoculation site (Spoel and Dong, 2008; Andolfi et al., 2011; Yang et al., 2015; Fonseca et al., 2018). Our results showed that the stomatal closure induced by these active agents were not affected by IAA and ZR (Figures 3, 4). The asexual stage of *B. dothidea* is *Fusicoccum aesculi*, which belong to the same genus with *Fusicoccum amygdale*, the latter producing Fusicoccin stimulating stomatal opening (Squire and Mansfield, 2010). Therefore, it is possible that *B*. *dothidea*-induced stomatal closure is also associated with hormone-like toxins and secondary metabolites released by *B*. *dothidea*. Determination of these active substances and the underlying physiological mechanism require further study.

### Absorption and transformation of light energy

Adversity stress may generate irreversible damage to plant cell structure and cause metabolic disorder (Flexas and Medrano, 2002). Plants are susceptible to biotic stress that can disrupt their photosynthetic apparatus (Júnior et al., 2017). The decreased photosynthetic pigments and chlorophyll fluorescence parameters were also in charge of the reduced *P*_n_ in *B*. *dothidea*-inoculated plants (Figures 6–8). Environmental stress can affect the photosynthetic pigments metabolism of plants. Leaf pathogen infection can decline chlorophyll content, and hinder light energy absorption and electron transfer, thus suppressing the photosynthetic carbon assimilation (Mandal et al., 2009). *Seiridium cardinal*-infected cypress needles showed decreased content of chlorophyll and carotenoids (Muthuchelian et al., 2005). Compared to the control plants, poplar stems inoculated with *B*. *dothidea* had lower content of Chl a, Chl b, total Chl, and Car at 6 and 14 dpi in distal leaves (Figures 6A–D). In *B*. *dothidea*-inoculated plants, the chlorophyll a/b ratio was reduced at 6 and 14 dpi, indicating Chl b proportion increased in total Chl, which is conducive to efficient utilization of low light (Figure 6E). By absorbing excess light energy and quenching active oxygen, carotenoids help protect the photosynthetic apparatus (Chi et al., 2015). It is possible that *B*. *dothidea*-induced quenching of active oxygen is partly responsible for the simultaneous decrease of Car and Chl/Car (Figures 6D,F). Meanwhile, Car’s descent speed was much slower than Chl’s degradation rate, which might play a crucial role in light protection (Figure 6F). Several lines of evidence indicate that *B*. *dothidea* accelerates the degradation of photosynthetic pigments or inhibits their synthesis. Inoculating poplar stems with *B*. *dothidea* reduced photosynthetic pigments in distal leaves, causing *P*_n_ to decline. These results were further supported by the positive correlation between Chl and *P*_n_ (Figure 8A).

Chlorophyll fluorescence can give a good indication of the activity of the photosynthetic apparatus (Björkman and Demmig, 1987; Krause and Weis, 1991; Watling et al., 2000). *F*_v_/*F*_m_ can reflect the energy conversion efficiency of PSII reaction center, and this parameter will drop under environmental stress (Neil, 2008). For example, *Melampsora medusae*, a fungus that causes leaf rust, caused a striking reduction of *F*_v_/*F*_m_ in poplar trees (Fermín et al., 2018). Besides the leaf pathogen, stem pathogens, such as *Seiridium cardinale*, also inhibited leaf photosystem II in cypress canker (Muthuchelian et al., 2005). In this context, as a result of *B*. *dothidea* inoculation on poplar stems, *F*_v_/*F*_m_ declined in distal leaves, which indicates that *B*. *dothidea* inhibited the conversion of light energy (Figure 7A). In response to adversity stress, both ETR and *Φ*_PSII_ were restrained at different levels, which affected photosynthetic carbon assimilation (Guo et al., 2016). In distal leaves, *B*. *dothidea* decreased *Φ*_PSII_ and ETR, which suggests that this fungus suppressed electron transport efficiency (Figures 7B,C). This conclusion was further supported by the positive correlation between *Φ*_PSII_ and *P*_n_, indicating that the decrease in *P*_n_ was due to the obstruction of photosynthetic electron transfer (Figure 8B). In PSII, *q*_P_ and NPQ are commonly used to measure light energy utilization and dissipation. The *q*_P_ of distal leaves was decreased across 2–14 dpi following *B*. *dothidea* inoculation (Figure 7D). However, NPQ was consistently higher than control across the detecting stage (Figure 7E). After *B*. *dothidea* inoculation, the distal leaves dissipated the excess light energy by heat dissipation to maintain the normal photosynthesis physiological process. According to these results, a decrease in photosynthetic pigments content and a blockage of electron transport were partially responsible for *B*. *dothidea* inoculation suppressing *P*_n_.

As shown above, *B*. *dothidea*-inoculated on stems suppressed the activity of PSII reaction center of distal leaves, leading to reduction in light energy capture by antenna pigments, thereby declining light energy utilization. In response to the reduced photochemical efficiency, PSII accumulated a large amount of light energy. Leaf NPQ levels were increased to dissipate excess light energy, thereby protecting the photosynthetic apparatus.

### Carbohydrate content

When fungi invade plant tissues, necrosis occurs, net *P*_n_ and carbon assimilation are reduced (Berger et al., 2007; Christian et al., 2008). Photosynthesis produces starch and sucrose, which are widely considered energy sources for metabolism. As a non-reducing sugar, sucrose exhibits a high level of solubility, resistance to degradation, and low viscosity (Patrick et al., 2013). Due to these advantages, sucrose has become a major long-distance transport product in the phloem (Patrick et al., 2013). In response to environmental stress, the stomata can regulate gas exchange and control the absorption of CO_2_ (Melotto et al., 2006; Lawson and Vialet-Chabrand, 2019). When *B*. *dothidea* was inoculated on poplar stems, stomatal closure of distal leaves occurred and CO_2_ uptake was reduced (Figures 3, 4). In spite of this, persistent stomatal closure results in reduced CO_2_ uptake for plants, affecting their photosynthetic productivity (O′Grady et al., 2013). In agreement with this conclusion, a reduction in photosynthetic pigments and chlorophyll fluorescence parameters resulted in a lower net *P*_n_ (Figures 1A, 6–8). Photosynthetic products will be reduced by the above results. Nevertheless, *B. dothidea* inoculation on poplar stems reduced leaf sucrose content at 6 dpi, but by 14 dpi, leaf sucrose content rose (Figure 9A). Throughout the observation period, *B. dothidea* inoculation increased leaf starch levels (Figure 9B). Previously, we demonstrated that cankers disrupt the downward transport of photosynthate (Li et al., 2019; Xing et al., 2020). In brief, starch and soluble sugar content were higher in the above regions of *B*. *dothidea* inoculation sites than below at 20, 25, 30 dpi during long-term inoculation (Xing et al., 2020).

Phloem girdling involves removing all the phloem, causing more damage to the stem. Instead of phloem girdling, *B*. *dothidea* inoculation treatments in this study were just scraped the barks of inoculation sites gently without damaging the cambial, xylem, and most of the phloem tissue, resulting in less possibility switched to serious injury in short-term. Phloem girdling prevents the downward movement of starch, which builds up in leaves and inhibits photosynthesis through negative feedback (Paul and Pellny, 2003; Urban et al., 2004). Consequently, sucrose and starch accumulation in leaves might be due to the local phloem damage caused by *B*. *dothidea*, hindering the assimilates downwards (Figure 9). According to our results, *P*_n_ was not affected by the negative feedback mechanism in the early stage of *B*. *dothidea* inoculation, but whether it works during the later stage remains to be verified.

## Conclusion

In this study, *B*. *dothidea* inhibited the leaf photosynthesis in the early stage of inoculation (2–14 dpi). Physiological mechanisms are as follows, (i) Decreasing *G*_s_, *C*_i_, and increasing *L*_s_ following *B. dothidea* inoculation, indicating that stomatal limitation was responsible for inhibiting *P*_n_. We observed stomatal closure in leaves leading to reduction in CO_2_ absorption, as expected. (ii) Inoculation of *B*. *dothidea* on stems affected the metabolism of distal leaves’ photosynthetic pigments. Deficiencies in chlorophyll and carotenoids hindered light energy absorption, conversion and electron transfer. (iii) The decreased *F*_v_/*F*_m_, *Φ*_PSII_, ETR, *q*_P_, and increased NPQ indicated that *B*. *dothidea* could partially close PSII reaction center on distal leaves, reducing light energy capturing capacity by photosynthetic pigments, thereby declining light energy utilization. Photosynthetic pigments suppression was also reflected in the chlorophyll fluorescence parameters. In combination, the above findings listed in i, ii, and iii inhibited photosynthetic carbon assimilation, thereby reducing photosynthetic products. However, due to the local damage caused by inoculation in phloem prevented the downward transport of assimilation products, leading to an accumulation of sucrose and starch in leaves.

## Data availability statement

The original contributions presented in this study are included in the article/supplementary material, further inquiries can be directed to the corresponding authors.

## Author contributions

JX, JZ, and YZ conceived and design the original research plans. YZ supervised the experiments. JX and ML performed most of the experiments. JL, WS, and PL provided technical assistance to JX and ML. JX designed the experiments and analyzed the data. JX conceived the project and wrote the article with contributions of all the authors. YZ and JZ supervised and complemented the writing. All authors have read and approved the manuscript.

## References

[B1] Andolfi A. Mugnai L. Luque J. Surico G. Cimmino A. Evidente A. (2011). Phytotoxins produced by fungi associated with grapevine trunk diseases. *Toxins* 3 1569–1605. 10.3390/toxins3121569 22295177 PMC3268457

[B2] Bari R. Jones J. D. (2009). Role of plant hormones in plant defence responses. *Plant Mol. Biol.* 69 473–488. 10.1007/s11103-008-9435-0 19083153

[B3] Berger S. Sinha A. K. Roitsch T. (2007). Plant physiology meets phytopathology: Plant primary metabolism and plant-pathogen interactions. *J. Exp. Bot.* 58 4019–4026. 10.1093/jxb/erm298 18182420

[B4] Bernal M. Verdaguer D. Badosa J. Abadía A. Llusià J. Peñuelas J. (2015). Effects of enhanced UV radiation and water availability on performance, biomass production and photoprotective mechanisms of *Laurus nobilis* seedlings. *Environ. Exp. Bot.* 109 264–275. 10.1016/j.envexpbot.2014.06.016

[B5] Biggs A. R. Davis D. D. Merrill W. (1983). Histopathology of cankers on *Populus* caused by *Cytospora chrysosperma*. *Can. J. Bot.* 61 563–574. 10.1139/b83-064

[B6] Björkman O. Demmig B. (1987). Photon yield of O2 evolution and chlorophyll fluorescence characteristics at 77 K among vascular plants of diverse origins. *Planta* 170 489–504. 10.2307/23379014 24233012

[B7] Blackman C. J. Creek D. Maier C. Aspinwall M. J. Drake J. E. Pfautsch S. (2019). Drought response strategies and hydraulic traits contribute to mechanistic understanding of plant dry-down to hydraulic failure. *Tree Physiol.* 39 910–924. 10.1093/treephys/tpz016 30865274

[B8] Blackman P. G. Davies W. J. (1984). Age-related changes in stomatal response to cytokinins and abscisic acid. *Ann. Bot.* 54 121–126. 10.1093/oxfordjournals.aob.a086765

[B9] Blatt M. R. (2000). Cellular signaling and volume control in stomatal movements in plants. *Annu. Rev. Cell. Dev. Bio.* 16 221–241. 10.1146/annurev.cellbio.16.1.221 11031236

[B10] Cerqueira A. Alves A. Berenguer H. Correia B. Gómez-Cadenas A. Diez J. J. (2017). Phosphite shifts physiological and hormonal profile of Monterey pine and delays *Fusarium circinatum* progression. *Plant Physiol. Bioch.* 114 88–99. 10.1016/j.plaphy.2017.02.020 28284060

[B11] Chi S. C. Mothersole D. J. Dilbeck P. Niedzwiedzki D. M. Zhang H. Qian P. (2015). Assembly of functional photosystem complexes in *Rhodobacter sphaeroides* incorporating carotenoids from the spirilloxanthin pathway. *BBA-Bioenergetics* 1847 189–201. 10.1016/j.bbabio.2014.10.004 25449968 PMC4331045

[B12] Christian C. Frank F. Karl-Heinz H. Rainer M. Wolfgang O. (2008). Photosynthetic and leaf water potential responses of Alnus glutinosa saplings to stem-base inoculation with *Phytophthora alni* subsp. *alni*. *Tree Physiol.* 28 1703–1711. 10.1093/treephys/28.11.1703 18765375

[B13] Czemmel S. Galarneau E. R. Travadon R. Mcelrone A. J. Cramer G. R. Baumgartner K. (2015). Genes expressed in grapevine leaves reveal latent wood infection by the fungal pathogen *Neofusicoccum parvum*. *PLoS One* 10:e0121828. 10.1371/journal.pone.0121828 25798871 PMC4370485

[B14] Farquhar G. D. Sharkey T. D. (1982). Stomatal conductance and photosynthesis. *Annu. Rev. Plant Physiol.* 33 317–345. 10.1146/annurev.pp.33.060182.001533

[B15] Fermín G. José G. J. Corina G. (2018). Plant-pathogen interactions: Leaf physiology alterations in poplars infected with rust (*Melampsora medusae*). *Tree Physiol.* 38 925–935. 10.1093/treephys/tpx174 29370416

[B16] Flexas J. Medrano H. (2002). Drought-inhibition of photosynthesis in C3 plants: Stomatal and non-stomatal limitations revisited. *Ann. Bot.* 89 183–189. 10.1093/aob/mcf027 12099349 PMC4233792

[B17] Fonseca S. Radhakrishnan D. Prasad K. Chini A. (2018). Fungal production and manipulation of plant hormones. *Curr. Med. Chem.* 25 253–267. 10.2174/0929867324666170314150827 28292238

[B18] Franziska E. Erica P. Heiko V. Louwrance P. W. Almuth H. Daniel V. (2018). Rust infection of black poplar trees reduces photosynthesis but does not affect isoprene biosynthesis or emission. *Front. Plant Sci.* 9:1733. 10.3389/fpls.2018.01733 30538714 PMC6277707

[B19] Gruber B. R. Kruger E. L. McManus P. S. (2012). Effects of cherry leaf spot on photosynthesis in tart cherry ‘Montmorency’ foliage. *Phytopathology* 102 656–661. 10.1094/PHYTO-12-11-0334 22667445

[B20] Guillermo M. M. Franco R. R. Pablo E. V. María C. N. Santiago J. M. (2021). Stem canker caused by *Phomopsis* spp. induces changes in polyamine levels and chlorophyll fluorescence parameters in pecan leaves. *Plant Physiol. Bioch.* 166 761–769. 10.1101/2021.03.04.433926 34217132

[B21] Guo Y. Y. Yu H. Y. Kong D. S. Yan F. (2016). Effects of drought stress on growth and chlorophyll fluorescence of *Lycium ruthenicum* Murr. seedling. *Photosynthetica* 54 524–531. 10.1007/s11099-016-0206-x

[B22] Gururani M. A. Venkatesh J. Tran L. S. (2015). Regulation of photosynthesis during abiotic stress-induced photo inhibition. *Mol. Plant.* 8 1304–1320. 10.1016/j.molp.2015.05.005 25997389

[B23] Hossain M. Veneklaas E. J. Hardy G. E. S. T. J. Poot P. (2019). Tree host-pathogen interactions as influenced by drought timing: Linking physiological performance, biochemical defence and disease severity. *Tree Physiol* 39 6–18. 10.1093/treephys/tpy113 30299517

[B24] Irving H. R. Gehring C. A. Parish R. W. (1992). Changes in cytosolic pH and calcium of guard cells precede stomatal movements. *Proc. Natl. Acad. Sci. U.S.A.* 89 1790–1794. 10.1073/pnas.89.5.1790 11607281 PMC48538

[B25] Júnior A. F. N. Ribeiro R. V. Appezzato-da-Glória B. Soares M. K. M. Rasera J. B. Amorim L. (2017). *Phakopsora euvitis* causes unusual damage to leaves and modifies carbohydrate metabolism in Grapevine. *Front. Plant Sci.* 8:1675. 10.3389/fpls.2017.01675 29018470 PMC5623187

[B26] Krause G. H. Weis E. (1991). Chlorophyll fluorescence and photosynthesis: The basics. *Annu. Rev. Plant Physiol.* 42 313–349. 10.1146/annurev.pp.42.060191.001525

[B27] Ku Y. S. Sintaha M. Cheung M. Y. Lam H. M. (2018). Plant hormone signaling crosstalks between biotic and abiotic stress responses. *Int. J. Mol. Sci.* 19:3206. 10.3390/ijms19103206 30336563 PMC6214094

[B28] Kunkel B. N. Harper C. P. (2018). The roles of auxin during interactions between bacterial plant pathogens and their hosts. *J. Exp. Bot.* 69 245–254. 10.1093/jxb/erx447 29272462

[B29] Lachenbruch B. Zhao J. P. (2019). Effects of phloem on canopy dieback, tested with manipulations and a canker pathogen in the *Corylus avellana/Anisogramma anomala* host/pathogen system. *Tree Physiol.* 39 1086–1098. 10.1093/treephys/tpz027 30938425

[B30] Lawson T. Vialet-Chabrand S. (2019). Speedy stomata, photosynthesis and plant water use efficiency. *New Phytol.* 221 93–98. 10.1111/nph.15330 29987878

[B31] Li P. Liu W. X. Zhang Y. N. Xing J. C. Li J. X. Feng J. X. (2019). Fungal canker pathogens trigger carbon starvation by inhibiting carbon metabolism in poplar stems. *Sci. Rep.* 9:10111. 10.1038/s41598-019-46635-5 31300723 PMC6626041

[B32] Major I. T. Nicole M. C. Duplessis S. Séguin A. (2010). Photosynthetic and respiratory changes in leaves of poplar elicited by rust infection. *Photosynth. Res.* 104 41–48. 10.1007/s11120-009-9507-2 20012201

[B33] Mandal K. Saravanan R. Maiti S. Kothari I. L. (2009). Effect of downy mildew disease on photosynthesis and chlorophyll fluorescence in *Plantago ovate* Forsk. *J. Plant Dis. Protect.* 116 164–168. 10.1007/BF03356305

[B34] Marsberg A. Kemler M. Jami F. Nagel J. H. Postma-Smidt A. Naidoo S. (2017). *Botryosphaeria dothidea*: A latent pathogen of global importance to woody plant health. *Mol. Plant Pathol.* 18 477–488. 10.1111/mpp.12495 27682468 PMC6638292

[B35] McLachlan D. H. Kopischke M. Robatzek S. (2014). Gate control: Guard cell regulation by microbial stress. *New Phytol.* 203 1049–1063. 10.1111/nph.12916 25040778

[B36] Mcpartland J. M. Schoeneweiss D. F. (1984). Hyphal morphology of *Botryosphaeria dothidea* in vessels of unstressed and drought-stressed stems of *Betula alba*. *Phytopathology* 74 358–362. 10.1094/Phyto-74-358

[B37] Melotto M. Underwood W. Koczan J. Nomura K. Sheng Y. H. (2006). Plant stomata function in innate immunity against bacterial invasion. *Cell* 126 969–980. 10.1016/j.cell.2006.06.054 16959575

[B38] Meng L. Mestdagh H. Ameye M. Audenaert K. Höfte M. Marie-Christine V. L. (2020). Phenotypic variation of *Botrytis cinerea* isolates is influenced by spectral light quality. *Front. Plant Sci.* 11:1233. 10.3389/fpls.2020.01233 32903526 PMC7438557

[B39] Moradi F. Ismail A. M. (2007). Responses of photosynthesis, chlorophyll fluorescence and ROS-scavenging systems to salt stress during seedling and reproductive stages in rice. *Ann. Bot.* 99 1161–1173. 10.1093/aob/mcm052 17428832 PMC3243573

[B40] Muthuchelian K. Porta N. L. Bertamini M. Nedunchezhian N. (2005). Cypress canker induced inhibition of photosynthesis in field grown cypress (*Cupressus sempervirens* L.) needles. *Physiol. Mol. Plant Pathol.* 67 33–39. 10.1016/j.pmpp.2005.08.007

[B41] Neil R. B. (2008). Chlorophyll Fluorescence: A probe of photosynthesis *in vivo*. *Annu. Rev. Plant Bio.* 59 89–113. 10.1146/annurev.arplant.59.032607.092759 18444897

[B42] O′Grady A. P. Mitchell P. J. M. Pinkard E. A. Tissue D. T. (2013). Thirsty roots and hungry leaves: Unravelling the roles of carbon and water dynamics in tree mortality. *New Phytol.* 200 294–297. 10.1111/nph.12451 24050630

[B43] Parke J. L. Oh E. Voelker S. Hansen E. M. Buckles G. Lachenbruch B. (2007). *Phytophthora ramorum* colonizes tanoak xylem and is associated with reduced stem water transport. *Phytopathology* 97 1558–1567. 10.1094/PHYTO-97-12-1558 18943716

[B44] Patrick J. W. Botha F. Birch R. G. (2013). Metabolic engineering of sugars and simple sugar derivatives in plants. *Plant Biotechnol. J.* 11 142–156. 10.1111/pbi.12002 23043616

[B45] Paul M. J. Pellny T. K. (2003). Carbon metabolite feedback regulation of leaf photosynthesis and development. *J. Exp. Bot.* 54 539–547. 10.1093/jxb/erg052 12508065

[B46] Raish S. Saeed E. E. Sham A. Alblooshi K. El-Tarabily K. A. Abuqamar S. F. (2020). Molecular characterization and disease control of stem canker on royal poinciana (*Delonix regia*) caused by *Neoscytalidium dimidiatum* in the United Arab Emirates. *Int. J. Mol. Sci.* 21 6000–6029. 10.3390/ijms21031033 32033175 PMC7036867

[B47] Rohrs-Richey J. K. Mulder C. P. H. Winton L. M. Stanosz G. (2011). Physiological performance of an Alaskan shrub (*Alnus fruticosa*) in response to disease (*Valsa melanodiscus*) and water stress. *New Phytol.* 189 295–307. 10.1111/j.1469-8137.2010.03472.x 20868393

[B48] Ruehr N. K. Gast A. Weber C. Daub B. Arneth A. (2016). Water availability as dominant control of heat stress responses in two contrasting tree species. *Tree Physiol.* 36 164–178. 10.1093/treephys/tpv102 26491055

[B49] Saiki S. T. Ishida A. Yoshimura K. Yazaki K. (2017). Physiological mechanisms of drought-induced tree die-off in relation to carbon, hydraulic and respiratory stress in a drought-tolerant woody plant. *Sci. Rep.* 7:2995. 10.1038/s41598-017-03162-5 28592804 PMC5462810

[B50] Savi T. Casolo V. Borgo A. D. Rosner S. Torboli V. Stenni B. (2019). Drought-induced dieback of *Pinus nigra*: A tale of hydraulic failure and carbon starvation. *Conserv. Physiol.* 7:coz012. 10.1093/conphys/coz012 31198559 PMC6541882

[B51] Schroeder J. I. Kwak J. M. Allen G. J. (2001). Guard cell abscisic acid signalling and engineering drought hardiness in plants. *Nature* 410 327–330. 10.1038/35066500 11268200

[B52] Slippers B. Wingfield M. J. (2007). *Botryosphaeriaceae* as endophytes and latent pathogens of woody plants: Diversity, ecology and impact. *Fungal Biol. Rev.* 21 90–106. 10.1016/j.fbr.2007.06.002

[B53] Spoel S. H. Dong X. (2008). Making sense of hormone crosstalk during plant immune responses. *Cell Host Microbe* 3 348–351. 10.1016/j.chom.2008.05.009 18541211

[B54] Squire G. R. Mansfield T. A. (2010). The action of fusicoccin on stomatal guard cells and subsidiary cells. *New Phytol.* 73 433–440. 10.1111/j.1469-8137.1974.tb02120.x

[B55] Suzuki N. Rivero R. M. Shulaev V. Blumwald E. Mittler R. (2014). Abiotic and biotic stress combinations. *New Phytol.* 203 32–43. 10.1111/nph.12797 24720847

[B56] Urban L. Léchaudel M. Lu P. (2004). Effect of fruit load and girdling on leaf photosynthesis in *Mangifera indica* L. *J. Exp. Bot.* 55 2075–2085. 10.1093/jxb/erh220 15310823

[B57] Verma V. Ravindran P. Kumar P. P. (2016). Plant hormone-mediated regulation of stress responses. *BMC Plant Biol.* 16:86. 10.1186/s12870-016-0771-y 27079791 PMC4831116

[B58] Watling J. R. Press M. C. Quick W. P. (2000). Elevated CO_2_ induces biochemical and ultrastructural changes in leaves of the C4 cereal sorghum. *Plant Physiol.* 123 1143–1152. 10.1104/pp.123.3.1143 10889263 PMC59077

[B59] Xing J. C. Li P. Zhang Y. N. Li J. X. Liu Y. Lachenbruch B. (2020). Fungal pathogens of canker disease trigger canopy dieback in poplar saplings by inducing functional failure of the phloem and cambium and carbon starvation in the xylem. *Physiol. Mol. Plant* 112:101523. 10.1016/j.pmpp.2020.101523

[B60] Yang J. Zhang J. Wang Z. Zhu Q. Wang W. (2001). Hormonal changes in the grains of rice subjected to water stress during grain filling. *Plant Physiol.* 127 315–323. 10.1104/pp.127.1.315 11553759 PMC117987

[B61] Yang Y. X. Ahammed G. J. Wu C. Fan S. Y. Zhou Y. H. (2015). Crosstalk among jasmonate, salicylate and ethylene signaling pathways in plant disease and immune responses. *Curr. Protein Pept. Sci.* 16 450–461. 10.2174/1389203716666150330141638 25824390

[B62] Zhu X. Wang L. Yang R. Han Y. Hao J. Liu C. (2019). Effects of exogenous putrescine on the ultrastructure of and calcium ion flow rate in lettuce leaf epidermal cells under drought stress. *Hortic. Environ. Biotechnol.* 60 479–490. 10.1007/s13580-019-00151-7

